# Endoplasmic Reticulum Stress Contributes to Helicobacter Pylori VacA-Induced Apoptosis

**DOI:** 10.1371/journal.pone.0082322

**Published:** 2013-12-13

**Authors:** Yuko Akazawa, Hajime Isomoto, Kayoko Matsushima, Tsutomu Kanda, Hitomi Minami, Naoyuki Yamaghchi, Naota Taura, Ken Shiozawa, Ken Ohnita, Fuminao Takeshima, Masayuki Nakano, Joel Moss, Toshiya Hirayama, Kazuhiko Nakao

**Affiliations:** 1 Department of Gastroenterology and Hepatology, Nagasaki University Hospital, Nagasaki, Japan; 2 Department of Bacteriology, Institute of Tropical Medicine, Nagasaki University, Nagasaki, Japan; 3 Cardiovascular and Pulmonary Branch, NHLBI, National Institutes of Health, Bethesda, Maryland, United States of America; University of Washington, United States of America

## Abstract

Vacuolating cytotoxin A (VacA) is one of the important virulence factors produced by *H. pylori*. VacA induces apoptotic cell death, which is potentiated by ammonia. VacA also causes cell death by mitochondrial damage, via signaling pathways that are not fully defined. Our aim was to determine whether endoplasmic reticulum (ER) stress is associated with VacA-induced mitochondrial dysfunction and apoptosis. We found that C/EBP homologous protein (CHOP), a key signaling protein of ER stress-induced apoptosis, was transcriptionally up-regulated following incubation of gastric epithelial cells with VacA. The effect of VacA on CHOP induction was significantly enhanced by co-incubation with ammonium chloride. Phosphorylation of eukaryotic initiation factor 2 (eIF2)-alpha, which is known to occur downstream of the ER stress sensor PKR-like ER-localized eIF2-alpha kinase (PERK) and to regulate CHOP expression, was also observed following incubation with VacA in the presence of ammonium chloride. Knockdown of CHOP by siRNA resulted in inhibition of VacA-induced apoptosis. Further studies showed that silencing of the PERK gene with siRNA attenuated VacA-mediated phosphorylation of eIF2-alpha, CHOP induction, expression of BH3-only protein Bim and Bax activation, and cell death induced by VacA with ammonium chloride, indicating that ER stress may lead to mitochondrial dysfunction during VacA-induced toxicity. Activation of ER stress and up-regulation of BH3-only proteins were also observed in human *H. pylori-*infected gastric mucosa. Collectively, this study reveals a possible association between VacA-induced apoptosis in gastric epithelial cells, and activation of ER stress in *H. pylori-*positive gastric mucosa.

## Introduction

Infection with *Helicobactor pylori* (*H. pylori*) may result in chronic gastritis, gastric ulcer, and gastric cancer [1]–[4]. Vacuolating cytotoxin A (VacA) is one of the major toxins produced by *H. pylori* that may trigger molecular changes in gastric epithelial cells. [3]–[10]. Secreted by the bacteria as a ∼88-kDa single polypeptide [11], VacA contains amino-terminal 33.4-kDa (p33) and carboxy-terminal 54.8-kDa (p55) domains [11]–[13]. It is believed that p33 is responsible for the assembly of VacA into stable hexamers that form an ion channel, which is required for VacA-induced toxicity, while p55 is responsible for VacA binding to cells [10], [12], [14], [15]. Following acid activation, a p33-dependent, anion-selective channel is formed, leading to VacA internalization and association with endosomal membranes [13]. It has been proposed that internalized VacA incorporated into channels accelerates the turnover of endosomal V-ATPases by augmenting the permeability of the endosomal membrane to anions, leading to the accumulation of osmotically active species such as NH_4_^+^ [13], [16]. This event is believed to induce an osmotic imbalance involving late endosomes that provokes vacuolation. In this regard, VacA-induced vacuolation is inhibited by the V-ATPase activity inhibitor, Bafliomycin A1 [17]. In contrast, weak bases including NH_4_Cl that can be produced by the high urease activity of *H. pylori* significantly potentiates VacA-mediated vacuole formation in cultured cells [5], [18], [19].

VacA is also known to cause apoptosis in gastric epithelial cells. It is now accepted that VacA targets mitochondria to mediate cell death [6], [10], [12], [20], [21]. The unresolved question has been whether VacA induces cytochrome *c* release by directly or indirectly targeting mitochondria. Domanska *et al.* showed that VacA forms ion channels on mitochondria in a p33-dependent manner [12], whereas Yamasaki and others suggested that VacA causes cytochrome *c* release indirectly by activating the pro-apoptotic Bcl-2 family protein Bax [20]. The mechanism by which VacA induces Bax activation is not fully understood. Notably, both VacA-induced vacuolation and mitochondrial dysfunction were significantly enhanced by NH_4_Cl, while ammonia *per se* did not induce significant cell injury [5], [20], [22]. According to these studies, NH_4_Cl is likely not necessary for VacA to initiate apoptosis but it significantly increases VacA-induced mitochondrial dysfunction and cytotoxicity [5]. Enhancement of both vacuolation and mitochondrial dysfunction by NH_4_Cl is inhibited by ion channel blockers, suggesting that membrane channel formation is required for both activities [17]. On the other hand, a study employing AZ-521 cells and MKN 28 cells demonstrated that apoptosis by neither VacA alone nor VacA in combination with NH_4_Cl was attenuated by Bafilomycin A1 [5], [20]. Thus at least in selected cell lines, NH_4_Cl may potentiate VacA-mediated apoptosis via an unknown mechanism that is independent of vacuolation.

Endoplasmic reticulum (ER) plays a role in critical cellular functions by controlling protein folding and trafficking [23], [24]. Failure of the ER's capacity to resolve stress results in induction of the unfolded protein response (UPR), which interacts with other stress signaling pathways including those involved in inflammation and cell death [24], [25]. The ER stress transducers in mammalian cells are PKR-like ER-localized eukaryotic initiation factor 2 (eIF2)-α kinase (PERK), inositol-requiring enzyme 1(IRE-1), and Activating transcription factor 6 (ATF6) [23], [26], [27]. In unstressed cells, these proteins are retained in an inactive conformation via their association with the ER-resident chaperone protein, glucose-regulated protein 78/immunoglobulin-heavy-chain-binding protein (GRP78) [23], [26]. When unfolded proteins increase in the ER, GRP78 is released from PERK, ATG-6 and IRE-1, thereby activating the three ER stress sensors [23], [24]. ER stress, especially activation of PERK, leads to induction of nuclear C/EBP-homologous protein (CHOP) via phosphorylation of Eukaryotic Initiation Factor (eIF2)-α [28], [29]. CHOP has been implicated as a key mediator of ER stress-induced cell death in diverse pathological conditions including gastric epithelial cell damage [30]–[34], and is known to activate proteins that mediate mitochondrial dysfunction [25], [32], [34], [35]. Of note, CHOP has been reported to activate pro-apoptotic BH3-only proteins including Bcl-2 interacting mediator of cell death (Bim) and p53 up-regulated modulator of apoptosis (PUMA) [35]. These BH3-only proteins usually monitor cellular wellbeing but they participate in promoting Bax activation to initiate mitochondrial cell death when activated by cytotoxic signals [36]. However, contribution of ER stress and its downstream effectors during VacA-induced cell injury remains to be defined. To gain further mechanistic insight into VacA-induced mitochondrial dysfunction and cell death stimulated by ammonia, we investigated the effects of VacA and ammonia on the PERK- and CHOP-signaling pathway and its potential role in the activation of Bax, PARP cleavage, and cell death.

## Materials and Methods

### Cells

A human gastric cancer cell line, AZ-521 (Culture Collection of Health Science Resource Bank, Japan Health Science Foundation, Tokyo, Japan), was used in the study. Cells were grown in Eagle's minimal essential medium (Sigma) containing 10% fetal calf serum (Invitrogen, Carlsbad, CA) under a 5% CO_2_ atmosphere at 37°C.

### VacA Preparation and Treatment

The toxin-producing *H. pylori* strain ATCC 49503 was the source of VacA, with purification by a modification of our published procedure [37]. In brief, after growth of *H. pylori* in Brucella broth containing 0.1% β-cyclodextrin at 37°C for 3–4 days with vigorous shaking in a controlled microaerobic atmosphere of 10% O_2_ and 10% CO_2_, VacA was precipitated from the culture supernatant with 50% saturated ammonium sulfate. Precipitated proteins were then dialyzed against RX buffer (10 mM KCl, 0.3 mM NaCl, 0.35 mM MgCl_2_, and 0.125 mM EGTA in 1 mM HEPES, pH 7.3) and applied to an anti-VacA-specific immunoglobulin G (IgG) antibody column equilibrated with RX buffer. After washing the column with RX buffer, VacA was subsequently eluted with 50 mM glycine-HCl buffer (pH 1.0), which was promptly neutralized with 1 M Tris–HCl (pH 10). After gel filtration on Superose 6HR 10/30 equilibrated with TBS buffer (60 mM Tris–HCl buffer, pH 7.7, containing 0.1 M NaCl), purified VacA was concentrated and stored at −20°C (200 µg/ml). VacA concentration was measured using a bead enzyme-linked immunosorbent assay method. To activate VacA, 0.2 v/v 300 mM HCl was added to VacA preparations, which were then incubated for 10 min at room temperature, and then neutralized with the same volume of 300 mM NaOH. The cells were treated with 120 nM of VacA [20]. In selected experiments, cells were treated with VacA in the presence of 5 mM NH_4_Cl, which was similar to the concentrations observed in human *H. pylori*-associated gastritis [38].

### Assay for Vacuolating Activity

Neutral red uptake into vacuoles was quantified as previously described [37]. Cells were incubated with 50 µl of 0.05% neutral red in PBS containing 0.3% bovine serum albumin. Cells were then washed three times with 0.1 ml of PBS containing 0.3% bovine serum albumin. After addition of 0.1 ml of 70% ethanol in water containing 0.4% HCl, absorbance at 540 nm (*A*_540_) was measured.

### Quantitation of Apoptosis

Cells were stained with 5 µg/ml of 4′,6-diamidine-2′-phenylindole dihydrochloride (DAPI) for 30 min at 37°C and visualized under fluorescence microscopy (Nikon Eclipse TE200; Nikon, Tokyo, Japan). Apoptotic cells were quantified by counting 100 random cells per study. Cells with the characteristic nuclear changes of chromatin condensation and nuclear fragmentation were considered apoptotic. In addition, apoptosis was also confirmed biochemically by immunobloting for cleaved PARP (catalog # 9541, Cell Signaling, Beverly, MA).

### Immunoblot Analysis

Whole cell lysates were directly lysed for 15 min. on ice using a commercially available cell lysis reagent from Thermo Scientific (Rockford, IL). For CHOP protein analysis, nuclear extracts from whole cell lysates were obtained using nuclear extraction reagent (Thermo Scientific, Rockford, IL) following manufacturer's instructions. Samples containing 50 µg of protein were resolved by 4–15% SDS-PAGE, transferred to nitrocellulose membranes, followed by incubation overnight with primary antibodies at a dilution of 1∶1000. The primary antibodies used are as follows; Mouse anti-Bax **(**catalog # sc-7480), rabbit anti-Bad (catalog # sc-7869), rabbit anti-Bik (catalog # sc-10770), mouse anti-CHOP (catalog # sc-7351), and anti-Lamin B (catalog # sc-6216**)** antibodies were from Santa Cruz (Santa Cruz, CA). Goat anti-Bid antibodies were from R and D Systems (catalog # BAF860), rabbit anti-PUMA antibodies (catalog # 23404) were from Rockland (Gilbertsville, PA), and rabbit anti-Bak antibodies(catalog # 06-536) were from Upstate (Billerica, MA). Rabbit anti-Bim, rabbit anti-PERK (catalog # 3129S), rabbit anti-Cleaved PARP (catalog# 5625S) rabbit anti-eIF2-α (catalog # 9722), and rabbit anti-phospho-eIF2-α (catalog # 9721) antibodies were from Cell Signaling Technology (Beverly, MA). On the following day, membranes were incubated with appropriate horseradish peroxidase-conjugated secondary antibodies (Biosource International, Camarillo, CA) at a dilution of 1∶3000 for 2 hr. Bound antibody was incubated with chemiluminescent substrate (SuperSignal® West Pico Chemiluminescent, Thermo Scientific, Rockford, IL) for 5 min and was visualized with a chemiluminescent imaging system (FluorChem® FC2, Alpha Innotech, San Leandro, CA). In selected experiments, density of bands was analyzed using Image J software (National Institutes of Health, Bethesda, MD) in order to quantify the results.

### RNA Isolation and Quantitative Reverse-transcriptase Polymerase Chain Reaction

Biopsy specimens were placed immediately into 1 mL of RNA later (Applied Biosystems, Foster City, CA), followed by extraction of total RNA using a commercially available kit (GenElute™ Mammalian total RNA Miniprep Kits, Sigma-Aldrich, Munich, Germany). Total RNA was extracted from the cells and tissue using the kit mentioned above. RNA was quantified using Nonodrop-1000 spectrophotometer (Nanodrop Technologies, Wilmington, DE). RNA was reverse-transcribed into complementary DNA with random primers (Invitrogen, Grand Island, NY). Quantification of the complementary DNA template was performed with real-time PCR (LightCycler 480; Roche Applied Science) using SYBR green (Molecular Probes) as a fluorophore. PCR primers were as follows; for human CHOP, forward (5′-ATGGCAGCTGAGTCATTGCCTTTC-3′) and reverse (5′-AGAAGCAGGGTCAAGAGTGGTGAA-3′), for human PUMA forward (5′-GACGACCTCAACGCACAGTA-3′) and reverse (5′AGGAGTCCCATGATGAGATTGT-3′), and for human Bim forward (5′-AGATCCCCGCTTTTCATCTT-3′) and reverse (5′-TCTTGGGCGATCCATATCTC-3′). Primers for 18S ribosomal RNA (rRNA) were used (Ambion, Austin, TX) as an internal control. The relative mRNA expression levels are given as a ratio of target mRNA/18S rRNA per each sample.

### Small Interfering (si) RNA for PERK and CHOP

Small interfering RNA (siRNA) was employed to knockdown CHOP and PERK. CHOP targeting nucleotides, which included 4 different sequences, were obtained from Dharmacon (Lafayette, CO, CHOP siGENOME SMARTpool, catalog # M-004819-03-0005). PERK targeting nucleotides were obtained from SIGMA and Ambion. The target sequences were (PERK siRNA: 5′-CACAAACUGUAUAACGGUUUA-3′), and (PERK siRNA #2: 5′-GUGACGAAAUGGAACAAGA-3′), respectively. Briefly, cells were grown in 6-well plates and transiently transfected with siRNA using Lipofectamin RNAiMAX (Invitrogen Grand Island, NY). Cells were used for experiments 48 hr after transfection. Knock down of CHOP was confirmed by real-time PCR and knock down of PERK was assessed by immunoblotting.

### Assessment of Bax Activation

Immunocytochemistry of activated Bax was performed using mouse monoclonal anti-Bax (clone 6A7, 1∶400 dilution, Exalpha Biologicals, Watertown, MA) as previously described in detail [39]. Cells were imaged by confocal microscopy with excitation and emission wavelengths of 488 and 507 nm, respectively.

### Human Biopsy Samples

Patients who underwent upper gastrointestinal endoscopy from June 2007 to May 2011 were enrolled in the study. Upper gastrointestinal endoscopy was performed in Nagasaki University Hospital. Endoscopies were performed for medically indicated reasons. Endoscopies were not performed only for research. Written informed consent of the patient was obtained prior to enrollment, in agreement with the Helsinki Declaration. The study was reviewed and approved by an Ethics Committee of Nagasaki University Hospital (Office of Human Subjects Protection Registration number IORG0007678). Exclusion criteria were as follows: age <18 or >80 years, pregnancy, body mass index >30 kg/m^2^, diabetes mellitus, malignancies, renal impairment, systemic infection, liver diseases, drug addiction, alcohol abuse, use of medications effective against *H. pylori* during the preceding 3 months, and chronic corticosteroid or nonsteroidal anti-inflammatory drug use. During endoscopic examination of Japanese patients, 2 biopsy specimens were obtained from the gastric antrum along the lesser curvature. One of the samples was subjected to RNA isolation. The other specimen was fixed in 10% formalin and embedded in paraffin for histopathological examination. *H. pylori* status was assessed by the rapid urease test (Helicocheck, Otsuka Pharmaceutical, Tokushima, Japan) and histology with Giemsa staining. Patients were considered positive for *H. pylori* infection when at least one of these examinations yielded positive results. Patients were defined as *H. pylori*-negative if all test results were negative.

### Immunohistochemistry

Immunohistochemistry for GRP78 was performed with the streptavidin-biotin-peroxidase-complex method (Histofine SAB-PO® kits, Nichirei Co., Tokyo, Japan). Paraffin-embedded biopsy specimens were sliced into 5-µm-thick sections, deparaffinized, and rehydrated. After inhibition of endogenous peroxidase activity for 30 min with methanol containing 0.3% H_2_O_2_, the sections were reacted for 20 min with 5% albumin. Samples were subsequently incubated overnight at 4°C with anti-GRP78 antibody (sc-1050, Santa Cruz, CA), at a dilution of 1∶1000. On the following day, sections were washed in 0.01 M phosphate-buffered saline (PBS) and incubated for 20 min with 10 mg/mL biotinylated antiserum. After washing in PBS, the sections were incubated for 20 min with 100 µg/mL horseradish peroxidase-conjugated streptavidin and stained with 0.02% 3,3′-diaminobenzidine tetrahydrochloride in 0.05 M Tris–HCl buffer containing 0.03% H_2_O_2_. The sections were then washed in PBS and counterstained with hematoxylin.

### Statistical Analysis

All data represent at least three independent experiments and are expressed as means ± SE unless otherwise indicated. Differences between groups were compared by using either an unpaired two-tailed *t*-test, one-way ANOVA followed by post hoc test (Bonferroni method), or two-way ANOVA followed by post hoc test (Bonferroni method), as indicated in figure legends. *p* values <0.05 were considered statistically significant.

## Results

### VacA Induces ER Stress in AZ-521 Cells

**Figure 1:**
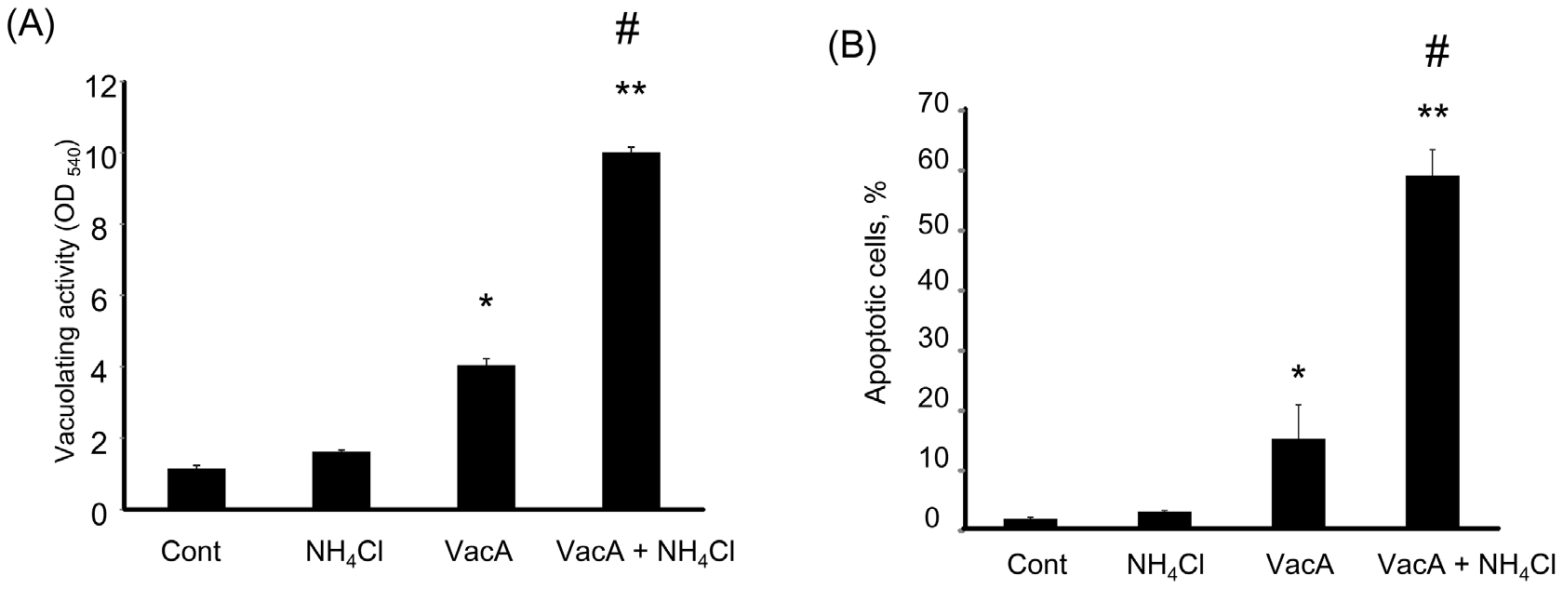
Effect of NH_4_Cl on VacA-induced vacuolation and apoptosis. (A) Cells were treated with either vehicle (control), 5 mM NH_4_Cl, 120 nM VacA, or 5 mM NH_4_Cl plus 120 nM VacA for 6 hr. Vacuolating activity was determined by neutral red uptake assay. The data represent the mean ± SEM for 4 independent experiments. Data was assessed by ANOVA followed by Bonferroni method. *p<0.01, control vs VacA-treated cells. **p<0.01,Vac A alone vs NH_4_Cl plus VacA-treated cells. # p<0.01, control vs NH_4_Cl plus VacA-treated cells. (B) Cells were treated with either vehicle (control), 5 mM NH_4_Cl, 120 nM VacA, or 5 mM NH_4_Cl plus 120 nM VacA for 24 hr. Apoptosis was assessed by morphological criteria after DAPI staining. The data represent the mean ± SEM for 3 independent experiments. Data was assessed by one-way ANOVA followed by Bonferroni method. *p<0.05, control vs VacA-treated cells. **p<0.01, VacA alone vs NH_4_Cl plus VacA-treated cells. # p<0.01, control vs NH_4_Cl plus VacA-treated cells.

**Figure 2:**
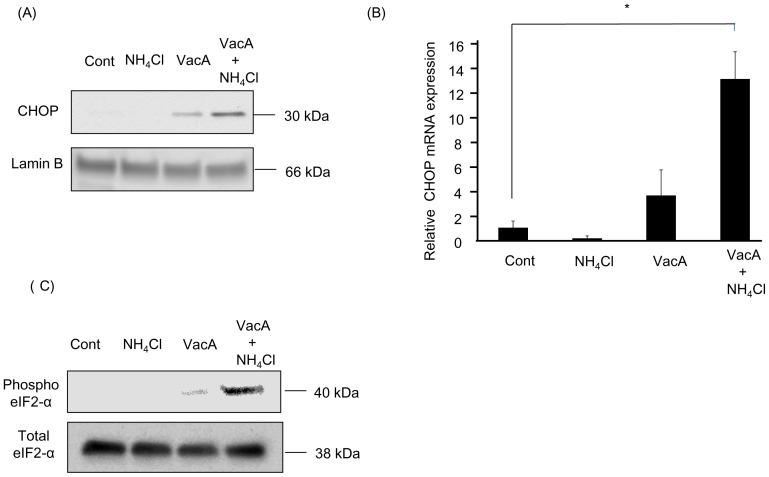
VacA induces CHOP expression and phosphorylation of eIF2-α. Cells were treated with either vehicle (control), 5 mM NH_4_Cl, 120 nM VacA, or 5 mM NH_4_Cl plus 120 nM VacA for 8 hr. (A) Nuclear extracts were obtained and resolved by SDS-PAGE. Proteins were identified by immunoblot analysis using anti-CHOP antibodies. Lamin B was used as a loading control. (B) The collected cells were subjected to real-time PCR. Relative mRNA fold induction was determined by normalization to 18S RNA. The data represent the mean ± SEM of 3 independent experiments, each performed in triplicate. Data was assessed by one-way ANOVA followed by Bonferroni method. *p<0.05, control vs VacA plus NH_4_Cl-treated cells. (C) Whole cell lysates were obtained and resolved by SDS-PAGE. Proteins were identified by immunoblot analysis using anti-phospho- and total- eIF2-α antibodies.

**Figure 3:**
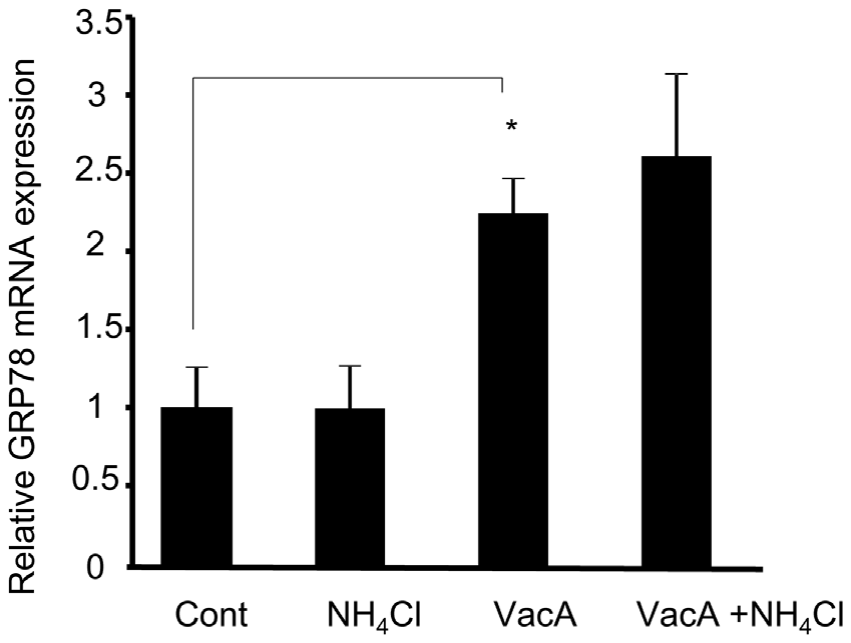
VacA treatment induces expression of GRP78 mRNA. Cells were treated with either vehicle (control), 5 mM NH_4_Cl, 120 nM VacA, or 5 mM NH_4_Cl plus 120 nM VacA for 8 hr. Expression of GRP78 was quantified by real-time PCR. The data represent the mean ± SEM for n = 3 studies. Data was assessed by one-way ANOVA followed by Bonferroni method. *p<0.05, control vs VacA-treated cells.

We initially tested whether NH_4_Cl increases vacuolation and apoptosis in AZ-521 cells. As previously reported, VacA alone triggered vacuolation in AZ-521 cells (Figure 1A) [18], which was increased in the presence of 5 mM NH_4_Cl (Figure 1A). VacA induced a mild increase in the number of apoptotic cells as confirmed by DAPI staining, whereas cell death was significantly enhanced when cells were incubated with VacA plus NH_4_Cl (Figure 1B). Next, we assessed whether VacA increases expression of CHOP, which has a pivotal role in ER-induced apoptosis. We incubated the cells with NH_4_Cl alone, VacA alone, or VacA plus NH_4_Cl and examined the CHOP protein by immunoblot analysis. CHOP protein was not present in untreated cells or NH_4_Cl-treated cells, whereas intoxication with VacA alone triggered weak expression of CHOP protein (Figure 2A). Of note, CHOP protein expression was augmented in cells treated with VacA plus NH_4_Cl (Figure 2A). Expression of CHOP mRNA was also increased by 13 fold in cells treated with VacA plus NH_4_Cl compared to untreated cells (Figure 2B), implying that CHOP was transcriptionally activated by apoptotic stimuli following ER stress. In fact, eIF2-α, which can cause transcriptional induction of CHOP downstream of ER-stress sensor PERK, was phosphorylated by incubation of cells with VacA plus NH_4_Cl (Figure 2C). These results suggested that induction of CHOP resulted from PERK activation via phosphorylation of eIF2-α. Expression of ER resident chaperone protein GRP78 mRNA was also significantly elevated in VacA-treated cells (Figure 3). Splicing of XBP-1 mRNA occurs downstream of the IRE-1 arm of ER stress; XBP-1 mRNA existed in both spliced and un-spliced form in untreated cells and this splicing pattern was not altered by VacA treatment (Figure S1). Up-regulation of ATF-6 mRNA was also not observed following VacA treatment (data not shown). Collectively, these results indicate that VacA in the presence of NH_4_Cl is capable of inducing ER stress, especially involving the downstream targets of PERK.

### PERK-CHOP Pathway Regulates VacA- Induced Apoptosis in AZ-521 Cells

**Figure 4:**
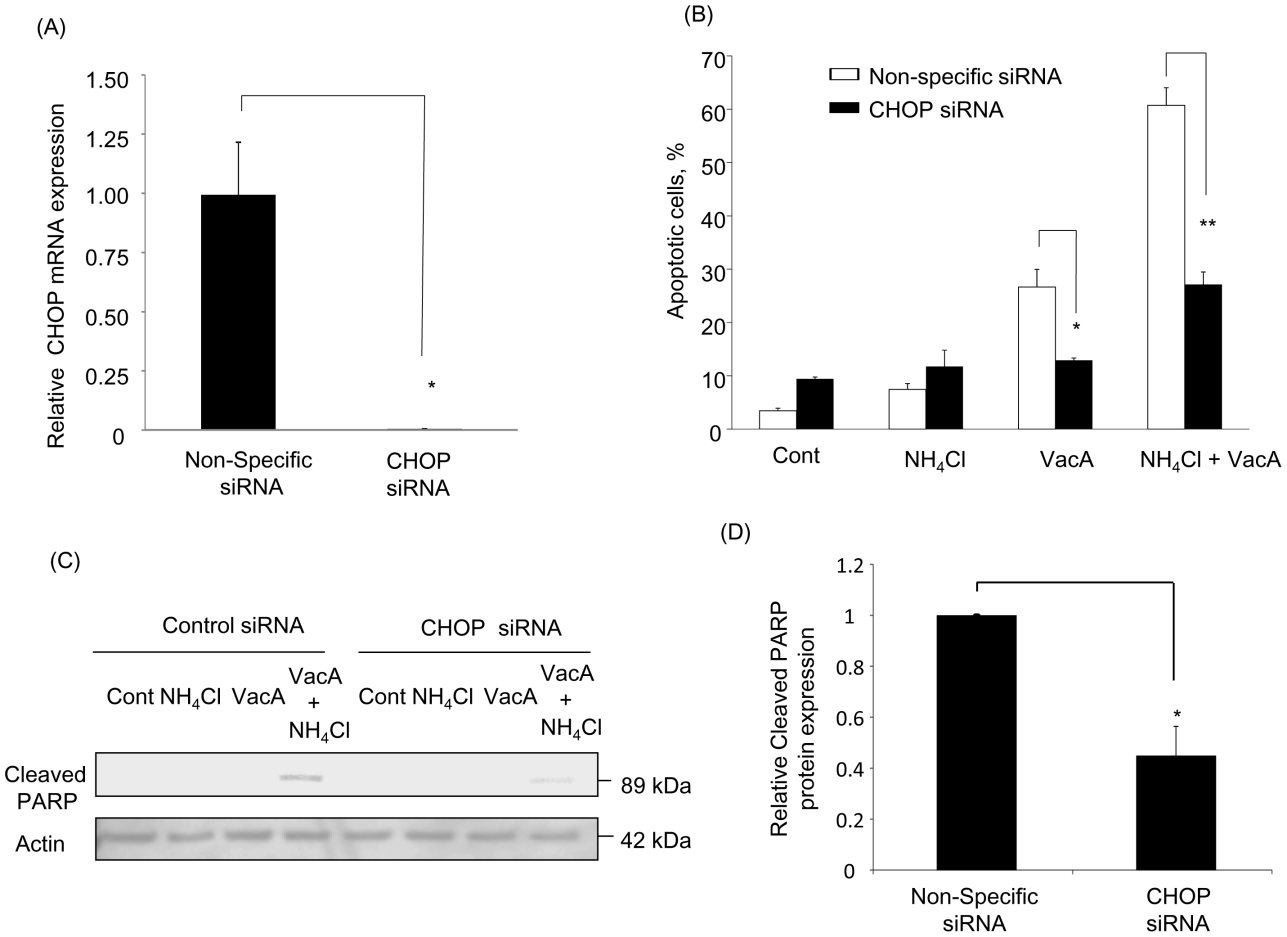
CHOP contributes to VacA-induced apoptosis. Cells were treated with either non-specific siRNA or CHOP siRNA for 48 hr. (A) CHOP mRNA expression was assessed by real-time PCR. Differences between groups were compared by using an unpaired two-tailed *t*-test. *p<0.05, non-specific siRNA-transfected cells vs CHOP siRNA-transfected cells. The data represent the mean ± SEM for n = 3 studies. (B) Cells were treated with either vehicle (control), 5 mM NH_4_Cl, 120 nM VacA, or 5 mM NH_4_Cl plus 120 nM VacA for 24 hr. Apoptosis was assessed by morphological changes after 30 min of DAPI staining. The data represent the mean ± SEM for n = 3 studies each performed in triplicate. Data was assessed by two-way ANOVA followed by Bonferroni method. *p<0.05, non-specific siRNA-transfected cells vs CHOP siRNA-transfected cells in VacA-treated cells. **p<0.01, non-specific siRNA-transfected cells vs CHOP siRNA-transfected cells in NH_4_Cl plus VacA-treated cells. (C) Cells were treated as indicated above. Cleaved PARP was assessed by immunoblotting. (D) After incubation with 5 mM NH_4_Cl plus 120 nM VacA for 24 hr, cells were collected and cleaved PARP was assessed by immunoblotting. Densitometry was performed and differences between groups were compared by using an unpaired two-tailed *t*-test.*p<0.05, non-specific siRNA vs CHOP siRNA transfected cells. The data represent the mean ± SEM for n = 3 studies.

**Figure 5:**
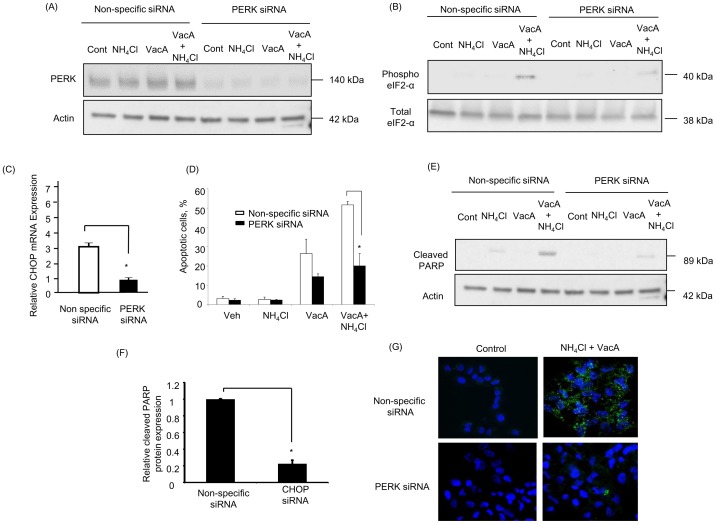
PERK contributes to CHOP activation and cell death. Cells were treated with either non-specific siRNA or PERK siRNA for 48 hr. (A) After treatment with either vehicle (control), 5 mM NH_4_Cl, 120 nM VacA, or 5 mM NH_4_Cl plus 120 nM VacA for 8 hr, PERK protein expression was assessed by immunoblotting. (B) After treatment with either vehicle (control), 5 mM NH_4_Cl, 120 nM VacA, or 5 mM NH_4_Cl plus 120 nM VacA for 8 hr, cells were lysed and were subjected to immunoblot analysis with anti-phospho-eIF2-α and anti-total eIF2-α antibodies. (C) After incubation with 5 mM NH_4_Cl plus 120 nM VacA for 8 hr, cells were collected and real-time PCR was performed to assess CHOP mRNA expression. The data represent the mean ± SEM for n = 3 studies. Differences between groups were compared by using an unpaired two-tailed *t*-test. *p<0.05 non-specific siRNA vs PERK siRNA. (D) Cells were treated with either vehicle (control), 5 mM NH_4_Cl, 120 nM VacA, or 5 mM NH_4_Cl plus 120 nM VacA for 24 hr. Apoptosis was assessed by morphological changes after 30 min of DAPI staining. Data was assessed by two-way ANOVA followed by Bonferroni method. *p<0.01, non-specific siRNA-transfected cells vs PERK siRNA-transfected cells. The data represent the mean ± SEM for n = 3 studies. (E) Cells were treated as indicated above. Cleaved PARP was assessed by immunoblotting. (F) After incubation with 5 mM NH_4_Cl plus 120 nM VacA for 24 hr, Expression of Cleaved PARP was assessed by immunoblotting followed by densitometry. Differences between groups were compared by using an unpaired two-tailed *t*-test. *p<0.05, non-specific siRNA vs PERK siRNA transfected cells. The data represent the mean ± SEM for n = 3 studies. (G) Cells were examined by immunofluorescence microscopy for Bax following the treatment with vehicle (control) or 5 mM NH_4_Cl plus 120 nM VacA for 16 hr. The primary antibody used for the study recognizes the N-terminal region of Bax, which is exposed upon activation. Green fluorescence shows activated Bax, whereas blue fluorescence indicates DAPI staining of nucleus. Data represent the results of 3 independent experiments.

Based on the above findings, we next examined whether activation of ER stress, particularly CHOP, is associated with VacA-induced apoptosis. AZ-521 cells were transfected with siRNA targeted to CHOP and inhibition of transcriptional expression was assessed by real-time PCR (Figure 4A). In response to treatment with both VacA alone and VacA plus NH_4_Cl, cells with knockdown of CHOP showed significant suppression of apoptosis (Figure 4B) and PARP cleavage (Figure 4C and D), indicating that CHOP participates in VacA-induced apoptosis. In subsequent studies, we tested whether induction of CHOP and its downstream apoptotic signaling was mediated by PERK. Indeed, PERK knockdown attenuated VacA-mediated phosphorylation of eIF2-α (Figure 5A and B) and CHOP mRNA expression (Figure 5C). More importantly, PERK knockdown decreased apoptosis (Figure 5D) and production of cleaved PARP caused by VacA treatment (Figure 5E and F). These results were further confirmed by employing an additional siRNA targeting PERK (Figure S2A, B, C, D). Next, we tested whether activation of ER stress occurs upstream or downstream of mitochondrial dysfunction. If ER stress occurs upstream of mitochondrial damage, knockdown of PERK should inhibit VacA-induced Bax activation. In fact, PERK siRNA attenuated Bax activation induced by VacA plus NH_4_Cl treatment (Figure 5G). These data suggest that upon VacA treatment, especially in the presence of NH_4_Cl, ER stress leads to PERK-mediated CHOP induction followed by mitochondrial dysfunction, leading to apoptotic cell death.

### VacA Induces Bim Expression in VacA-treated Cells Downstream of PERK

**Figure 6:**
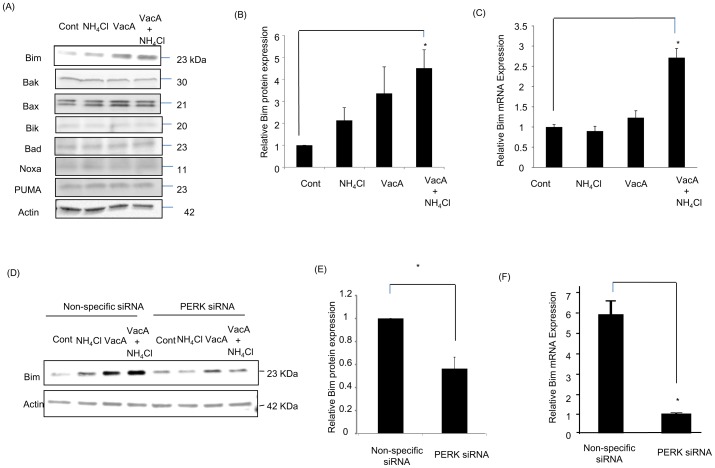
VacA induces expression of Bim via activation of ER stress. (A) Immunoblot analysis was employed for the assessment of pro-apoptotic-BH3-only proteins and Bcl-2 family proteins after the cells were treated with either vehicle (control), 5 mM NH_4_Cl, 120 nM VacA, or 5 mM NH_4_Cl plus 120 nM VacA for 8 hr. (B) Density of bands was analyzed to confirm increased expression of Bim protein in NH_4_Cl plus VacA-treated cells compared to control. Data was assessed by one-way ANOVA followed by Bonferroni method.*p<0.05, control vs VacA plus NH_4_Cl-treated cells. The data represent the mean ± SEM for n = 3 studies. (C) Cells were treated with either vehicle (control), 5 mM NH_4_Cl, 120 nM VacA, or 5 mM NH_4_Cl plus 120 nM VacA for 8 hr. Bim mRNA expression was assessed by real-time PCR. Data was assessed by one-way ANOVA followed by Bonferroni method. *p<0.05, control vs NH_4_Cl plus VacA-treated cells. The data represent the mean ± SEM for n = 3 studies. (D) After incubation with PERK siRNA or non-specific siRNA for 48 hr, cells were treated as above and were subjected for immunoblot analysis for Bim and actin. (E) After incubation with PERK siRNA or non-specific siRNA for 48 hr, cells were incubated with 5 mM NH_4_Cl plus 120 nM VacA for 8 hr. Expression of Bim was assessed by immunoblotting followed by densitometry. Differences between groups were compared by using an unpaired two-tailed *t*-test.*p<0.05, non-specific siRNA-transfected cells vs PERK siRNA-transfected cells. The data represent the mean ± SEM for n = 3 studies. (F) Cells were treated with either non-specific siRNA or PERK siRNA for 48 hr. After incubation with 5 mM NH_4_Cl plus 120 nM VacA for 8 hr, cells were collected and real-time PCR was performed to assess Bim mRNA expression. The data represent the mean ± SEM for n = 3 studies. Differences between groups were compared by using an unpaired two-tailed *t*-test. *p<0.05 non-specific siRNA vs PERK siRNA. Data represent the results of 3 independent experiments.

Since ER stress-mediated apoptosis can be executed via activation of BH3-only proteins [25], [40], we investigated whether BH3-only proteins are also up-regulated by VacA. We found that Bim protein was up-regulated in response to VacA treatment (Figure 6A and B), whereas other BH3-only proteins, such as PUMA, Noxa, Bad, Bik, and pro-apoptotic multidomain Bcl-2 proteins (Bak, Bax) were unchanged. Of note, Hrk and Bid were not detected in AZ-521 cells. We found that Bim mRNA was also up-regulated in cells treated with VacA plus NH_4_Cl (Figure 6C). Furthermore, VacA-induced expression of Bim protein (Figure 6D and E, Figure S2E and 2F) and mRNA (Figure 6F) were significantly attenuated by PERK siRNA, suggesting that, upon VacA treatment, Bim may be up-regulated downstream of ER stress pathways.

### Expression of ER Stress Markers and BH3-only Proteins in H. Pylori-infected Human Gastric Mucosa

**Figure 7:**
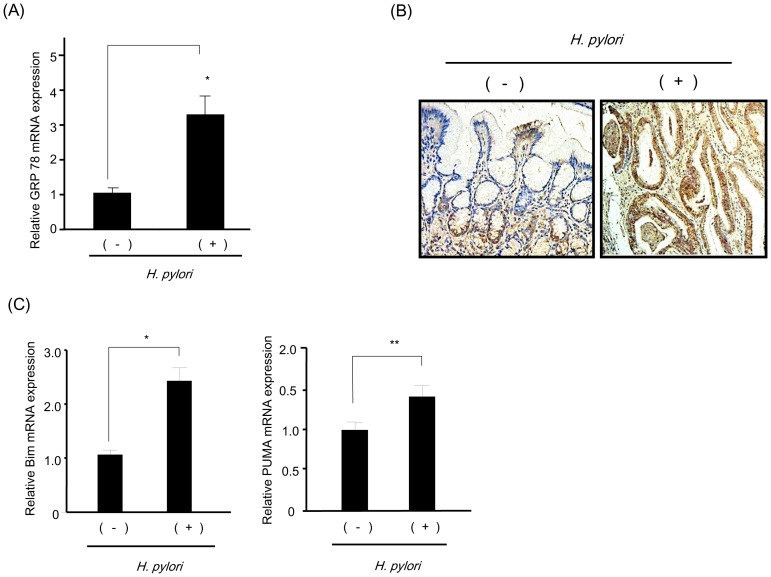
ER stress markers and BH3-only proteins are elevated in human *H. pylori-*infected mucosa. (A) mRNA was extracted from the biopsy specimen of *H. pylori*-negative and -positive gastric mucosa. Real-time PCR to quantify GRP78 mRNA expression was performed on the samples. Differences between groups were compared by using an unpaired two-tailed *t*-test. p<0.01, *H. pylori*-negative samples vs *H. pylori-*positive samples. *H. pylori* (−) n = 14, *H. pylori* (+) n = 22. (B) Immunohistochemistry of GRP78 in human gastric mucosa. GRP78 is identified by brown peroxidase staining. Right panel shows *H. pylori*-infected gastric mucosa (strong-positive GRP78 signal throughout the epithelium) and left panel shows healthy *H. pylori*-negative gastric mucosa (a lower GRP78 signal). Original magnification was 40×. (C) mRNA was extracted from the biopsy specimen of *H. pylori*-negative and -positive gastric mucosa. Real-time PCR of Bim (left panel) and PUMA (right panel) was performed on the samples. Differences between groups were compared by using an unpaired two-tailed *t*-test. *H. pylori* (−) n = 14, *H. pylori* (+) n = 22, *p<0.001, **p<0.05, H. pylori negative samples *vs H. pylori* positive samples.

To examine the above findings in *H. pylori*-infected human mucosa, we investigated the expression of ER stress markers. We found that expression of ER chaperone protein GRP78 mRNA was significantly elevated in *H. pylori-*infected samples compared to *H. pylori-*negative samples (Figure 7A), indicating activation of ER stress in *H. pylori*-infected mucosa. Immunohistochemistry for GRP78 showed that the protein was strongly positive in the epithelium in *H. pylori*-positive biopsy specimens, suggesting that activation of ER stress occurred in gastric epithelium (Figure 7B). In contrast, in the *H. pylori-*negative biopsy specimens, GRP78 was detected primarily in the crypts near the basement membrane and not in the upper layers of epithelium (Figure 7B). Expression of CHOP mRNA was unaltered in *H. pylori*-infected gastric mucosa (FigureS3). Next, mRNA expression of BH3-only proteins in *H. pylori*-infected and uninfected human mucosa was compared. We found that Bim and PUMA mRNAs were significantly elevated in *H. pylori*-infected mucosa compared to uninfected controls (Figure 7C). Collectively, these results suggest that a group of ER stress markers and BH3-only proteins are up-regulated in *H. pylori* infected-gastric mucosa.

## Discussion

The principal findings of this study indicate the following: (i) VacA induces ER stress in gastric epithelial cells, especially in the presence of NH_4_Cl, (ii) VacA-induced apoptosis is at least in part, dependent on activation of CHOP-mediated intracellular signaling, (iii) ER stress is responsible for induction of pro-apoptotic BH3-only protein Bim as well as Bax activation, and (iv) ER stress markers and BH3-only proteins are up-regulated in *H. pylori* infected-human gastric mucosa.

Apoptosis in response to *H. pylori* infection may play a role not only in gastric injury, but also in development of gastric atrophy and cancer. VacA, one of the most important virulence factors of *H. pylori,* induces vacuolation and apoptosis in gastric epithelial cells. Mitochondria serve as a target of VacA during apoptosis. The mechanism(s) underlying VacA effects on the mitochondria are unclear. It has been proposed that mitochondrial membrane potential is lost because of Bax induction, although the pathway leading to Bax activation has not been defined [13], [20], [41]. Others have suggested that loss of the membrane potential results from translocation of VacA to the mitochondria [12], [42]. The current study indicates that VacA induces ER stress in AZ-521 cells, consistent with previous reports demonstrating that other bacterial virulence factors including Shigella toxins and lipopolysaccharide provoke ER stress [43], [44]. Of note, some viruses and bacterial virulence factors trigger CHOP activation [44]–[46], implying that CHOP may participate in the pathogenesis of a number of infectious diseases. The significance of CHOP in facilitating ER stress-induced cell death has been well documented, for instance, macrophages from *CHOP* knockout mice are more resistant to apoptosis induced by thapsigargin, an activator of ER stress [31]; further, indomethacin-induced apoptosis is attenuated in cultured guinea-pig gastric mucosal cells expressing a dominant-negative form of CHOP [30]. Fibroblasts from *Bax* ^−/−^ *Bak* ^−/−^ mice are resistant to thapsigargin-induced apoptosis, indicating a pivotal role for these pro-apoptotic Bcl-2 family proteins in ER stress-mediated cell death [47]. Consistent with these reports, our study showed that VacA-mediated activation of CHOP, at least in part, contributes to apoptosis in gastric epithelial cells.

Studies have demonstrated that NH_4_Cl enhances VacA-induced Bax activation and cell death [8], [18]–[20]. In agreement, this study demonstrated that addition of 5 mM NH_4_Cl to incubation medium, a concentration often found in gastric juice of *H. pylori*-infected patients, significantly augmented VacA-mediated CHOP induction and apoptosis. Furthermore, CHOP was transcriptionally activated by PERK via phosphorylation of eIF2-α, which was also augmented by NH_4_Cl. Although it is currently unclear how NH_4_Cl increases VacA-induced ER stress, it is believed that VacA enters cells independent of ammonia [19], therefore, the effect of NH_4_Cl on apoptosis probably does not result from increased VacA entry into cells. We also note that the effect of NH_4_Cl on enhancing VacA-mediated ER stress and apoptosis may not be directly linked to enhanced vacuolation since CHOP induction upon incubation with VacA plus NH_4_Cl was not inhibited by Bafilomycin A1 (data not shown). These data support the prior conclusions that cytochrome *c* release and vacuolation are independent cellular outcomes of VacA treatment while both of these actions can be potentiated by the presence of NH_4_Cl in the culture medium [17], [20]. Our data showed that some ER stress markers including ATF-6 up-regulation and enhanced XBP-1 splicing were not altered by VacA treatment, suggesting that VacA in combination with NH_4_Cl does not broadly activate ER stress signals but rather specifically enhances PERK-CHOP signaling pathways. Interestingly, we did not observe increased expression of CHOP in human *H. pylori*-infected samples. The following possible scenarios are considered: persistent sub-lethal ER stress in human chronic gastritis caused by *H. pylori* might allow the cells to adapt for survival by retaining a minimal level of CHOP *in vivo*. In this respect, we observed up-regulation of cytoprotective GRP78 in VacA-treated cells as well as in human gastric mucosa infected by *H. pylori*. This ER-chaperone protein plays a critical role in a UPR-protective response and its up-regulation is commonly used as a sentinel marker of ER stress in various pathologic conditions [48]. Therefore, gastric cells may try to adapt to a toxic environment and UPR by increasing the content of chaperone proteins including GRP78. If ER homeostasis is not restored, apoptotic signals can be executed by activation of the PERK-CHOP pathway. Additional studies are necessary to test this hypothesis.

ER stress-mediated signaling pathways are coupled to activation of several death signaling molecules, which promote mitochondrial dysfunction [25]. Functionally, CHOP is known to up-regulate the BH3-only proteins Bim and PUMA and induce Bax activation [34], [35]. The role of BH3-only proteins in VacA-mediated apoptosis has not been explored, but VacA was reported to reduce expression of anti-apoptotic Bcl-2 proteins that antagonize the activation of BH3-only proteins [49], and Wei *et al.* have shown that BH3-only proteins are up-regulated in *H. pylori*-infected gastric epithelial cells [50]. In the current study, we found elevation of Bim and PUMA mRNA in human *H. pylori-*positive gastric mucosa, as well as transcriptional up-regulation of Bim in AZ-521 cells treated with VacA plus NH_4_Cl. Since knockdown of PERK decreased VacA-mediated Bim mRNA expression, activation of Bim likely occurred downstream of ER stress. Although further investigations are required to identify precisely how VacA-induced ER stress leads to activation of BH3-only proteins and Bax, it was recently reported that CHOP potentially cooperates with FOXO3a in neuronal cells to regulate ER stress-induced Bim expression [35]. Thus, it would be intriguing to investigate if FOXO3a also modulates VacA mediated-apoptosis in gastric epithelial cells.

In summary, the present study demonstrates that PERK-mediated CHOP up-regulation contributes to VacA-triggered Bax activation and apoptosis in gastric epithelial cells. Future research involving mouse models may lead to a better understanding of the role of ER stress in the pathogenesis of *H. pylori*-induced gastric mucosal injury.

## Supporting Information

Figure S1 **Cells were treated with either vehicle (control), 5 mM NH_4_Cl, 120 nM VacA, or 5 mM NH_4_Cl plus 120 nM VacA for 8 hr.** Cells were lysed and mRNA was collected. XBP-1 cDNA was amplified by PCR, following 3 hr incubation with restriction enzyme Pst-1. Unspliced form of XBP-1 demonstrates 290-bp and 183-bp products whereas spliced form shows a single 473-bp product. Data represent the results of 3 independent experiments.
(TIF)

Figure S2 **Cells were treated with either non-specific siRNA or PERK siRNA (PERK siRNA #2) for 48 hr.** (A) Knockdown of PERK was assessed by immunoblotting. (B) Cells were treated with either vehicle (control), 5 mM NH_4_Cl, 120 nM VacA, or 5 mM NH_4_Cl plus 120 nM VacA for 24 hr. Apoptosis was assessed by morphological changes after 30 min of DAPI staining. Data was assessed by two-way ANOVA followed by Bonferroni method. *p<0.01, non-specific siRNA-transfected cells vs PERK siRNA-transfected cells. The data represent the mean ± SEM of n = 3 studies. (C) Cells were treated as indicated above. Cleaved PARP was assessed by immunoblotting. (D) Cells were treated with 5 mM NH_4_Cl plus 120 nM VacA for 24 hr. Cleaved PARP was assessed by immunoblotting followed by densitometry. *p<0.05, non-specific siRNA vs PERK siRNA#2-transfected cells. Differences between groups were compared by using an unpaired two-tailed *t*-test. The data represent the mean ± SEM of n = 3 studies. (E) Cells were treated with either vehicle (control), 5 mM NH_4_Cl, 120 nM VacA, or 5 mM NH_4_Cl plus 120 nM VacA for 16 hr. Expression of Bim was assessed by immunoblotting. (F) Cells were treated with 5 mM NH_4_Cl plus 120 nM VacA for 8 hr. Expression of Bim was assessed by immunoblotting followed by densitometry. Differences between groups were compared by using an unpaired two-tailed *t*-test. *p<0.05, non-specific siRNA vs PERK siRNA#2-transfected cells.
(TIF)

Figure S3 **(A) mRNA was extracted from biopsy specimens of** ***H. pylori*** **-negative and -positive gastric mucosa.** Real-time PCR for CHOP mRNA expression was performed on the samples.
(TIF)
