# Supplementary figures and images for: Endoplasmic Reticulum Stress Contributes to Helicobacter Pylori VacA-Induced Apoptosis
Source: PLoS One. 2013 Dec 13;8(12):e82322. doi: 10.1371/journal.pone.0082322 (PMC3862672; doi:10.1371/journal.pone.0082322)

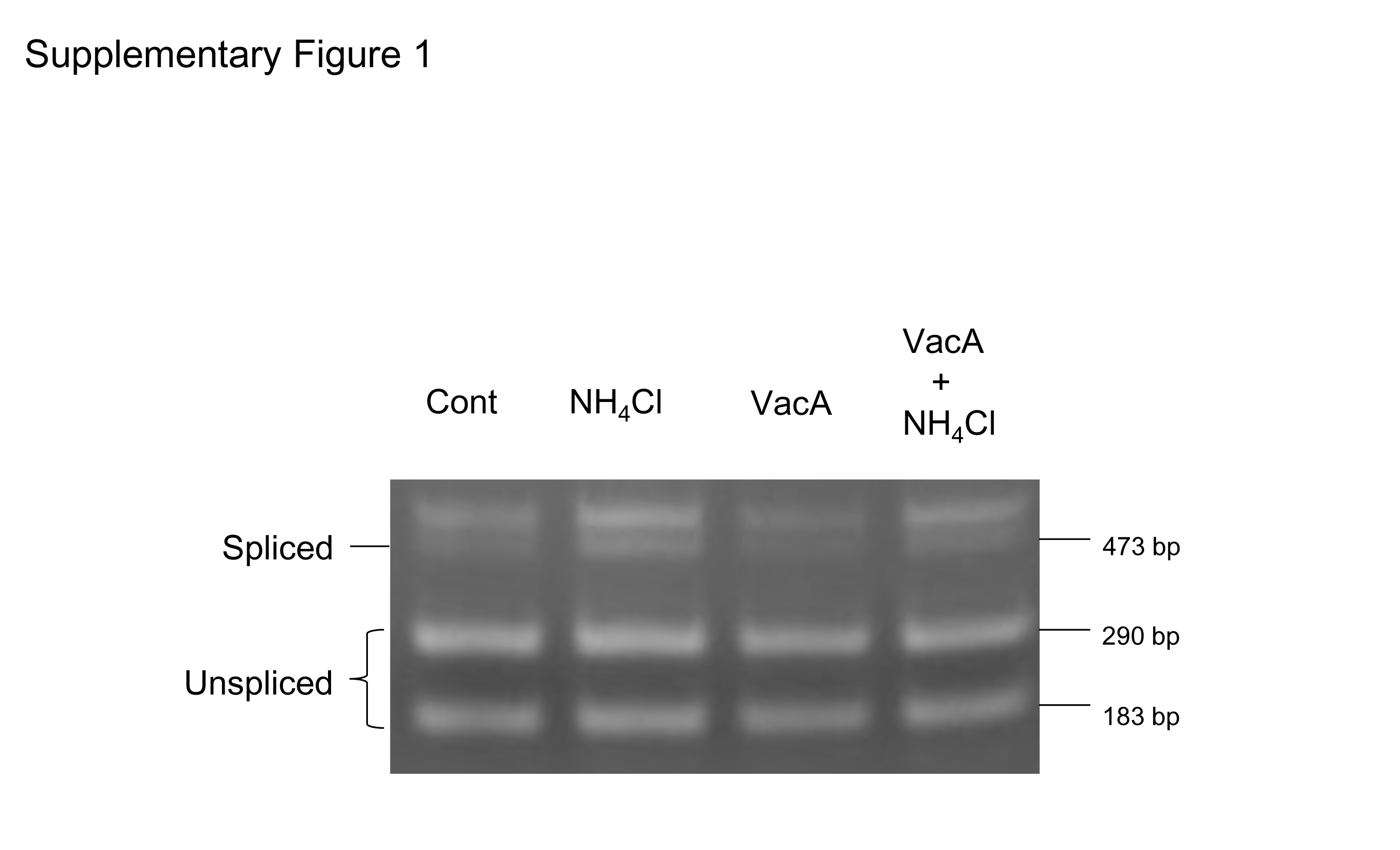

Supplement: Figure S1 — Cells were treated with either vehicle (control), 5 mM NH4Cl, 120 nM VacA, or 5 mM NH4Cl plus 120 nM VacA for 8 hr. Cells were lysed and mRNA was collected. XBP-1 cDNA was amplified by PCR, following 3 hr incubation with restriction enzyme Pst-1. Unspliced form of XBP-1 demonstrates 290-bp and 183-bp products whereas spliced form shows a single 473-bp product. Data represent the results of 3 independent experiments. (TIF) [file pone.0082322.s001.tif]

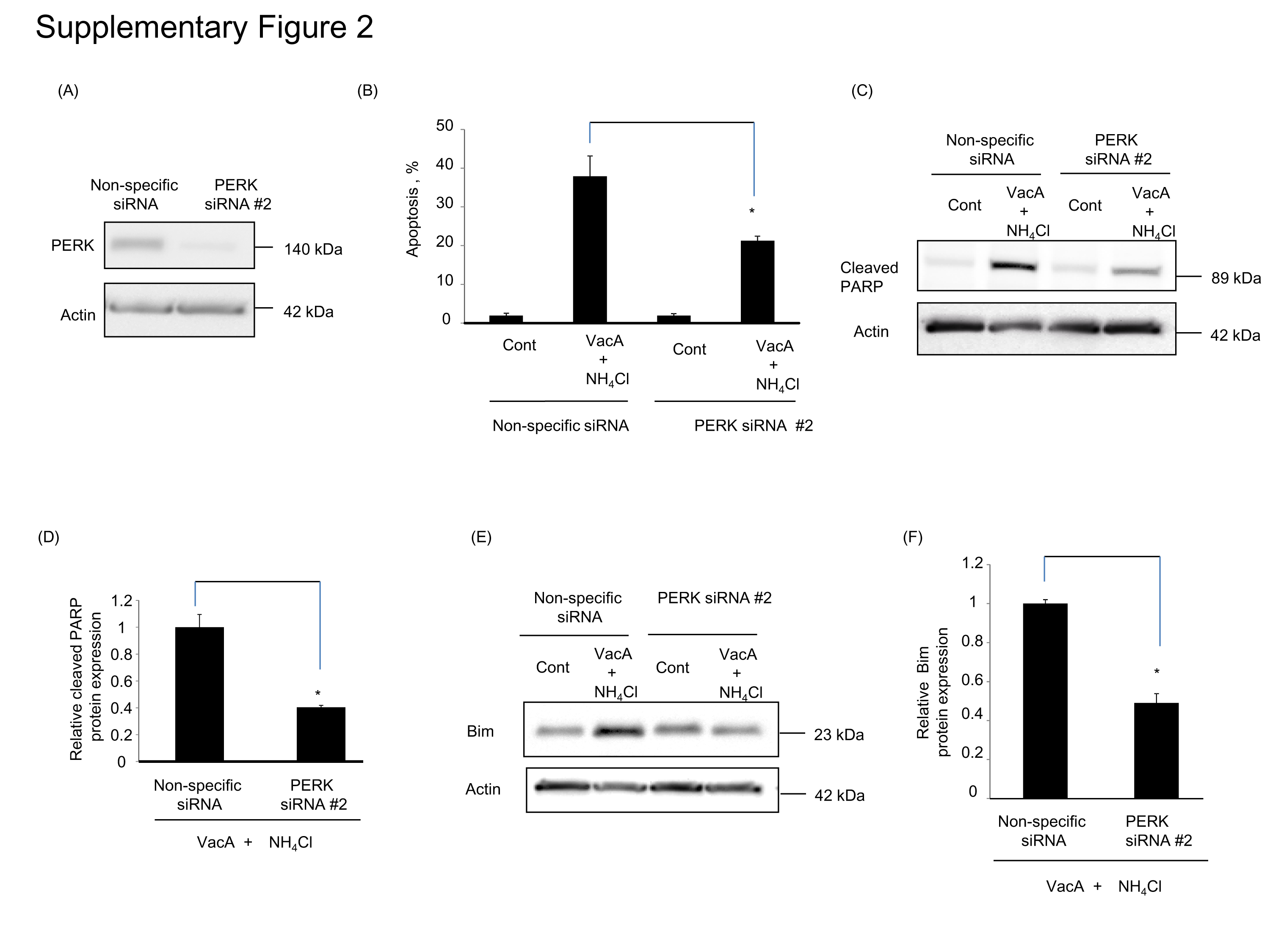

Supplement: Figure S2 — Cells were treated with either non-specific siRNA or PERK siRNA (PERK siRNA #2) for 48 hr. (A) Knockdown of PERK was assessed by immunoblotting. (B) Cells were treated with either vehicle (control), 5 mM NH4Cl, 120 nM VacA, or 5 mM NH4Cl plus 120 nM VacA for 24 hr. Apoptosis was assessed by morphological changes after 30 min of DAPI staining. Data was assessed by two-way ANOVA followed by Bonferroni method. *p<0.01, non-specific siRNA-transfected cells vs PERK siRNA-transfected cells. The data represent the mean ± SEM of n = 3 studies. (C) Cells were treated as indicated above. Cleaved PARP was assessed by immunoblotting. (D) Cells were treated with 5 mM NH4Cl plus 120 nM VacA for 24 hr. Cleaved PARP was assessed by immunoblotting followed by densitometry. *p<0.05, non-specific siRNA vs PERK siRNA#2-transfected cells. Differences between groups were compared by using an unpaired two-tailed t-test. The data represent the mean ± SEM of n = 3 studies. (E) Cells were treated with either vehicle (control), 5 mM NH4Cl, 120 nM VacA, or 5 mM NH4Cl plus 120 nM VacA for 16 hr. Expression of Bim was assessed by immunoblotting. (F) Cells were treated with 5 mM NH4Cl plus 120 nM VacA for 8 hr. Expression of Bim was assessed by immunoblotting followed by densitometry. Differences between groups were compared by using an unpaired two-tailed t-test. *p<0.05, non-specific siRNA vs PERK siRNA#2-transfected cells. (TIF) [file pone.0082322.s002.tif]

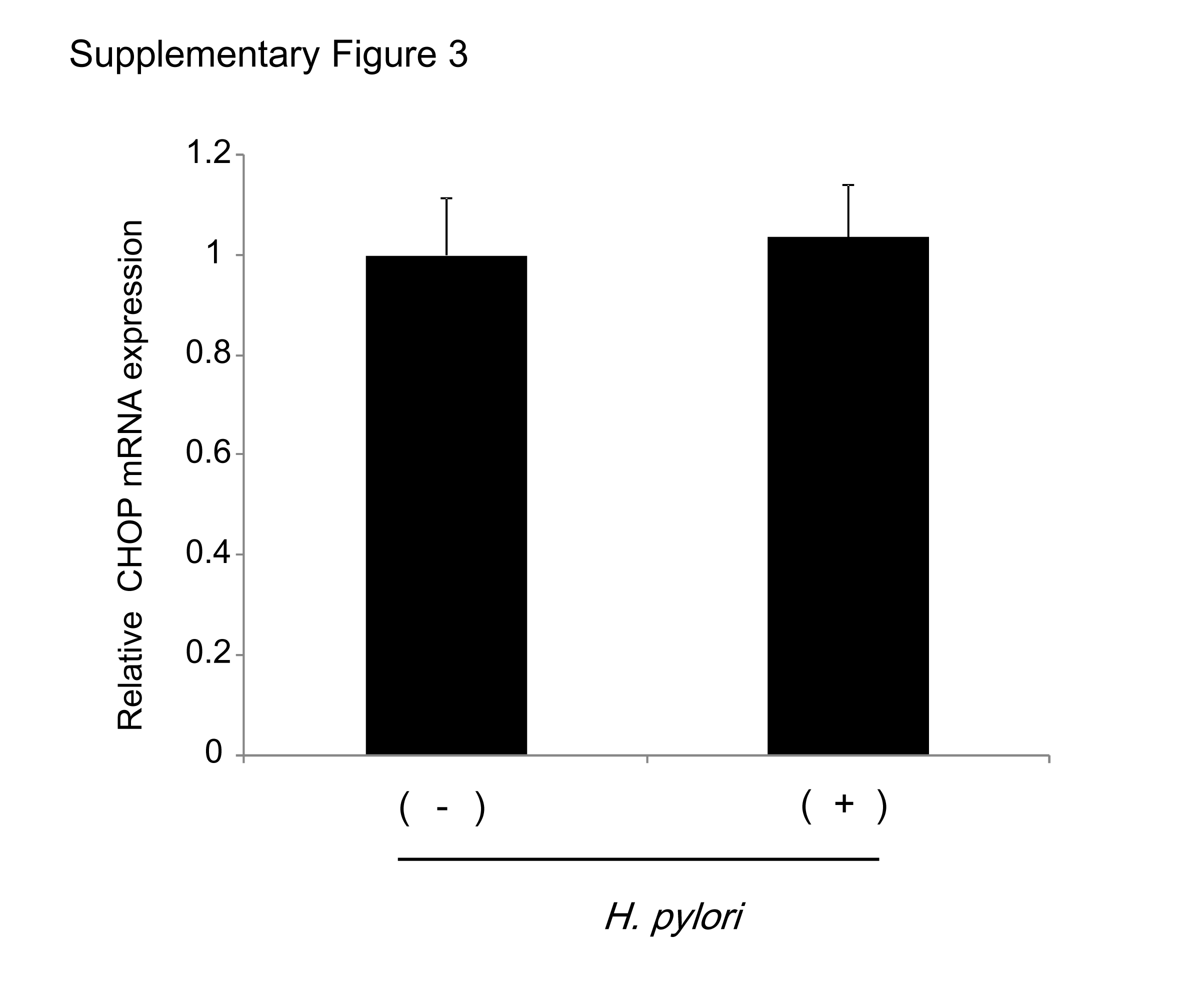

Supplement: Figure S3 — (A) mRNA was extracted from biopsy specimens of H. pylori -negative and -positive gastric mucosa. Real-time PCR for CHOP mRNA expression was performed on the samples. (TIF) [file pone.0082322.s003.tif]
